# Following the MAP for Improved Kidney Function in Hepatorenal Syndrome

**DOI:** 10.34067/KID.0000000000000118

**Published:** 2023-04-27

**Authors:** Matthew R. Thau, Pavan K. Bhatraju

**Affiliations:** 1 Division of Pulmonary, Critical Care and Sleep Medicine, University of Washington, Seattle, Washington; 2 Sepsis Center of Research Excellence (SCORE-UW), School of Medicine, University of Washington, Seattle Washington

**Keywords:** acute kidney failure

Hepatorenal syndrome type-1 (HRS-1) is a frequent cause of AKI in cirrhosis and is associated with significant mortality. The median survival in HRS-1 is 8–12 weeks, with a 30-day survival of 25%.^1^ Early recognition is essential to mitigate progression of renal dysfunction because the only definitive treatment for HRS-1 is liver transplantation, but HRS-1 is independently associated with increased mortality post-transplant. Moreover, elevated pretransplant serum creatinine and need for kidney replacement therapy is associated with nonresolution of HRS-1 after liver transplant.^2^ Treatment of HRS-1 often necessitates admission into the intensive care unit (ICU) for the administration of vasopressor support. In patients with HRS-1, the mean length of ICU stay is 10 days, with an average total hospital stay of 30.5 days.^3^ The diagnosis of AKI in patients with cirrhosis is independently associated with ICU admission, in addition to increased duration of hospital stay, development of multiple organ failure, and increased in-hospital mortality and 90-day mortality.^4^ Despite the significance of AKI in cirrhosis, several treatment questions remain, such as appropriate treatment parameters like mean arterial pressure (MAP) goals and urine output, to duration of treatment and weaning parameters, to more challenging questions such as when to consider renal replacement therapy or the effect of HRS-1 on liver transplantation evaluation, all leading to substantial resource allocation to these patients in the ICU.

In this issue of *Kidney360*, Velez and colleagues attempt to identify factors associated with improved kidney function in patients with HRS-1 and whether differences could be observed between norepinephrine and octreotide/midodrine. Of particular interest was determining whether kidney function improved with varying MAP increases of 5, 10, or 15 mm Hg or whether fixed titration parameters (such as an absolute targeted goal of 85 mm Hg) was sufficient. The retrospective cohort included 77 hospitalized patients with cirrhosis diagnosed with HRS-1 and treated with vasopressor therapy. The primary endpoint was defined as >30% reduction in serum creatinine on cessation of vasopressor support without the need for RRT or death. A significant reduction in serum creatinine was identified in patients with MAP increases of ≥15 mm Hg compared with those with increases of 5 or 10 mm Hg. No differences in serum creatinine was identified when patients were stratified by a fixed absolute parameter. The duration of vasopressor therapy needed to demonstrate evidence of therapeutic response also yielded interesting findings. Of the 25 patients who met the primary end point, 22 (88%) had improvements in serum creatinine within the first 48 hours, with 96% (24 total) by 72 hours, suggesting a fairly rapid response to vasopressor therapy in those who saw a significant improvement in kidney function.

These findings provide useful observations regarding the treatment goals and expectations for improved kidney function in patients with HRS-1. Patients with advanced cirrhosis typically have lower MAPs and decreased systemic vascular resistance at baseline, largely attributed to imbalance of compensatory homeostatic and vasoactive mechanisms.^5^ As a result, absolute parameter goals, such as a MAP of 85 mm Hg, may be difficult to achieve or require high doses of vasopressor support, whereas a targeted goal of ≥15 mm Hg is potentially a more reasonable approach. In addition, 96% of patients who experienced improvement in their kidney function demonstrated evidence within the first 72 hours of administration. This provides insight into a reasonable duration of time that can be expected to note if an improvement in kidney function is seen but also identifies an extremely at-risk population for morbidity and mortality among those who do not have any improvement within 72 hours.

This study does have several notable limitations. Only a third of the patients enrolled in the study met the primary study end point, limiting the studies' power to compare differences among the differing MAP parameters. In addition, drug administration was not uniformly executed as part of institutional protocols. In most instances, the goal of therapy was to achieve a rise in MAP of ≥5–15 mm Hg, an absolute MAP target of 85 mm Hg, or a range of absolute MAP of 80–85 or 85–90 mm Hg, making comparison difficult between the groups. Given the varying individual MAP response to vasopressor therapy, it is possible that the study identified a subgroup that was more responsive to vasopressor therapy and that this responsiveness provided therapeutic benefit, rather than the actual increase in MAP itself. Furthermore, the mean MAP of 70 seems high for this critically ill population, and an increase of 15 mm Hg in this instance interferes with the absolute targets that were specified. It would be interesting, for instance, to see whether an increase in MAP from 55 to 70 mm Hg was similarly efficacious.

In this study, 36% of the cohort received midodrine and octreotide, which has been shown to be less efficacious in the management of HRS-1 when compared with norepinephrine^6^ or terlipressin^7^ but remains the primary treatment outside of the ICU in the United States. Norepinephrine was the only vasopressor therapy available in the United States that had demonstrated improved kidney function in HRS^8^ before terlipressin was US Food and Drug Administration–approved for use in HRS in September 2022.^9^ Norepinephrine and terlipressin may have provided a more effective comparison, but terlipressin was notably associated with significant respiratory complications in a recent randomized trial.^10^ Of further note, serum creatinine is an imperfect marker for kidney function in patients with cirrhosis because it underestimates renal function due to reduced muscle mass, impaired hepatic production of creatine, and the increased tubular secretion of creatinine. The limitations of serum creatinine highlight the importance of identifying novel biomarkers of kidney function that may more accurately reflect changes in kidney function and facilitate earlier recognition of declining function before the development of HRS-1.

In conclusion, the results of this retrospective cohort study provide interesting insights into the MAP parameters and duration of time that should be targeted to maximize the likelihood for improved renal function. Future considerations might include a randomized trial that can better stratify MAP parameters to overcome the potential for confounding within the study or the administration of stable doses of norepinephrine to patients on lower acuity services outside of the ICU to determine whether a similar safety profile and improvement in kidney function can be identified.
